# The League of Mercy and Contributions from Hospital Funds to Medical Schools

**Published:** 1911-02-11

**Authors:** 


### THE LEAGUE OF MERCY AND CONTRIBUTIONS FROM HOSPITAL FUNDS TO MEDICAL SCHOOLS.

The question of the contribution from general hospital funds to medical school purposes has recently been the subject of correspondence in the *Times*. At the last meeting of Presidents of the League of Mercy the Duchess of Somerset raised the same question in regard to schools attached to hospitals receiving grants from the King's Fund and, through it, from the League of Mercy. Her Grace said : " She thought that the work of the League of Mercy should be entirely restricted to the relief of the sick and suffering, to which purpose the whole of the money collected should be applied. She had been asked to obtain an assurance on this point." Sir William Collins, one of the Hon. Secretaries, said, " The League had been assured by those responsible for the King's Fund that all moneys handed over by the League to the Fund and distributed to the hospitals went direct to the relief of the sick poor, and were not diverted to the medical schools."

We have caused inquiry to be made as to the action taken by the League of Mercy in regard to this question in order that no doubt may exist in the minds of its supporters and well-wishers, and we are informed that on April 29, 1908, a letter was sent by Mr. Beaumont, then secretary of the League, to the King's Fund, in which he said :—

I am desired by the Honorary Secretary of the League of Mercy to write to you with regard to a matter which bears very much on the work which our Society is doing. We are constantly asked whether the money collected by the League of Mercy goes in any way to the support of Medical Schools, there being a strong feeling among many of our workers that they do not wish to support Vivisection. At a meeting of the Executive Council of the League of Mercy held on March 16th, 1900, the following resolution was passed :—

> " That in the opinion of the Executive of the League of Mercy no moneys of the League should be assigned to any hospital which contributes to the support of Medical Schools or laboratories from its general fund for the relief of the patients."

The Honorary Secretaries of the League of Mercy understand that the King's Fund, after considering the report of Sir Edward Fry's Committee, passed a resolution which prescribes their present policy in the matter. May I ask you to be so kind as to inform me of the words of that resolution? This will enable me to answer the many questions which are addressed to me.

To this letter the following reply was sent :

King Edward's Hospital Fund for London."
7, Walbrook, E.C.
30th April, 1908.

H. E. Beaumont, Esq., Secretary, the League of Mercy.

Dear Sir,—In reply to your letter of April 29th, the following are the terms of the resolution passed by the King's Fund as a result of the report of Sir Edward Fry's Committee :

> " That the Executive Committee, having considered the recommendation of Sir Edward Fry's Committee (par. 27), advises the General Council to notify to the hospitals that no grants will be made from the King's Fund as from and after 1st January, 1906, to any hospitals which make payments to, or on account of, their medical schools out of general funds subscribed for the relief of the sick poor."

This resolution was duly notified to the hospitals on April 12th, 1905.

Yours faithfully,

(Signed) H. R. Maynard, Secretary.

We have been furnished with a copy of the letter which, on April 12th, 1905, was communicated to the hospitals concerned informing them of the policy adopted by the King's Fund on receiving the Report of Sir Edward Fry's Committee. It was as follows :—

King Edward's Hospital Fund for London.
81 Cheapside, E.C.
12th April, 1905.

The Hon. Secretaries beg to call your attention to the following Resolution, which was passed by the Executive Committee of this Fund on March 10th last and approved :—

> " That the Executive Committee, having considered the recommendation of Sir Edward Fry's Committee (par. 27), advises the General Council to notify to the Hospitals that no grants will be made from the King's Fund as from and after 1st January, 1906, to any Hospitals which make payments to, or on account of, their medical schools out of general funds subscribed for the relief of the sick poor."

The Executive Committee think it desirable to offer some explanatory remarks on the subject, which may be of assistance to the Committees of the Hospitals concerned.

Where the Hospital and School form a single institution, or where Subscribers have been in the habit of subscribing to the Hospital Funds knowing that grants are made from those Funds to the School, it will be necessary in future to keep separate accounts for the School, both in respect of receipts and expenditure. All money given to the Hospital, on and after January 1st, 1906, not specially earmarked by the donor, should be paid direct into the general account, and nothing should be paid out of the general account to, or on account of, the Medical School. It follows that the Medical School should be treated as a separate establishment, and that a sufficient payment for rent, repairs, insurance, and all value received from the Hospital by the Medical School should be made by the School to the Hospital.

In regard to interest on capital given to the Hospitals for the joint purposes of Hospital and School, an apportionment may be necessary, and the Executive Committee will be glad to receive and consider any scheme of apportionment which, if and when agreed to, would finally dispose of that part of the question as far as it relates to this Fund.

These observations apply only to Hospitals which desire to obtain grants from this Fund, and the conditions laid down are merely those which are precedent to grants being made.

To the Secretary, &c.

It will be apparent from these documents that the wishes of the League of Mercy and the views of supporters like the Duchess of Somerset have been amply met and safeguarded; and so long as this policy, deliberately arrived at, is pursued there need be no apprehension on the part of any of the workers for the League of Mercy that the funds they collect are used to support or encourage practices of which they may not approve.

